# 2-Phenyl-7-(4-pyridyl­methyl­amino)-1,2,4-triazolo[1,5-*a*][1,3,5]triazin-5(4*H*)-one^1^

**DOI:** 10.1107/S1600536810051032

**Published:** 2010-12-11

**Authors:** Anton V. Dolzhenko, Geok Kheng Tan, Anna V. Dolzhenko, Lip Lin Koh, Wai Keung Chui

**Affiliations:** a School of Pharmacy, Faculty of Health Sciences, Curtin University of Technology, GPO Box U1987, Perth 6845, Western Australia, Australia; b Department of Chemistry, Faculty of Science, National University of Singapore, 3 Science Drive 3, Singapore 117543, Singapore; c Perm State Pharmaceutical Academy, 2 Polevaya Street, Perm 614990, Russian Federation; d Department of Pharmacy, Faculty of Science, National University of Singapore, 18 Science Drive 4, Singapore 117543, Singapore

## Abstract

In the title compound, C_16_H_13_N_7_O, the 1,2,4-triazolo[1,5-*a*][1,3,5]triazine heterocyclic system is essentially planar (r.m.s. deviation = 0.0375 Å). The attached benzene ring lies almost in the mean plane of 1,2,4-triazolo[1,5-*a*][1,3,5]triazine [dihedral angle = 1.36 (23)°], while the pyridine ring is turned out of this plane by the amino­methyl bridge [dihedral angle = 69.22 (9)°]. The amino group H atom is involved in intra­molecular hydrogen bonding with a triazole N atom. In the crystal, mol­ecules are connected *via* C(=O)NH⋯N hydrogen bonds into *C*(11) chains parallel to [100]. The amino group H atom acts as a hydrogen-bond donor, forming an NH⋯O=C hydrogen bond with the carbonyl O atom, which links the mol­ecules into *C*(6) chains running along [011] and [01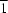].

## Related literature

For review on the synthesis and biological activity of 1,2,4-triazolo[1,5-*a*][1,3,5]triazines, see: Dolzhenko *et al.* (2006 ▶). For our work on the synthesis, crystal structure studies and biological activity of 1,2,4-triazolo[1,5-*a*][1,3,5]triazine, see: Dolzhenko *et al.* (2007*a* ▶,*b* ▶, 2008 ▶). For graph-set analysis of hydrogen bonding, see: Bernstein *et al.* (1995 ▶). For a related structure, see: Dolzhenko *et al.* (2011 ▶).
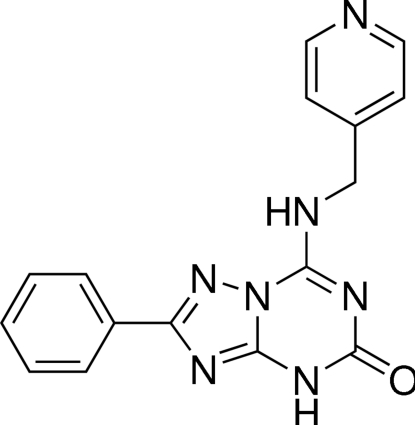


## Experimental

### 

#### Crystal data

C_16_H_13_N_7_O
*M* *_r_* = 319.33
Orthorhombic, 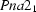

*a* = 22.142 (8) Å
*b* = 11.016 (4) Å
*c* = 5.992 (2) Å
*V* = 1461.5 (9) Å^3^
*Z* = 4
Mo *K*α radiation
μ = 0.10 mm^−1^
*T* = 100 K
0.58 × 0.36 × 0.04 mm

#### Data collection

Bruker SMART APEX CCD diffractometer
Absorption correction: multi-scan (*SADABS*; Sheldrick, 2001 ▶) *T*_min_ = 0.945, *T*_max_ = 0.996
9607 measured reflections
1830 independent reflections
1530 reflections with *I* > 2σ(*I*)
*R*_int_ = 0.088

#### Refinement

*R*[*F* ^2^ > 2σ(*F* ^2^)] = 0.067
*wR*(*F* ^2^) = 0.145
*S* = 1.19
1830 reflections
225 parameters
1 restraint
H atoms treated by a mixture of independent and constrained refinement
Δρ_max_ = 0.35 e Å^−3^
Δρ_min_ = −0.27 e Å^−3^

### 

Data collection: *SMART* (Bruker, 2001 ▶); cell refinement: *SAINT* (Bruker, 2001 ▶); data reduction: *SAINT*; program(s) used to solve structure: *SHELXS97* (Sheldrick, 2008 ▶); program(s) used to refine structure: *SHELXL97* (Sheldrick, 2008 ▶); molecular graphics: *SHELXTL* (Sheldrick, 2008 ▶); software used to prepare material for publication: *SHELXTL*.

## Supplementary Material

Crystal structure: contains datablocks I, global. DOI: 10.1107/S1600536810051032/hg2761sup1.cif

Structure factors: contains datablocks I. DOI: 10.1107/S1600536810051032/hg2761Isup2.hkl

Additional supplementary materials:  crystallographic information; 3D view; checkCIF report

## Figures and Tables

**Table 1:** Hydrogen-bond geometry (Å, °)

*D*—H⋯*A*	*D*—H	H⋯*A*	*D*⋯*A*	*D*—H⋯*A*
N5—H5*N*⋯N7^i^	0.90 (4)	1.89 (4)	2.782 (5)	173 (4)
N6—H6*N*⋯O1^ii^	0.95 (6)	1.96 (5)	2.735 (5)	137 (4)
N6—H6*N*⋯N1	0.95 (6)	2.31 (5)	2.813 (5)	112 (4)

Symmetry codes: (i) 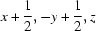; (ii) 
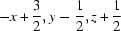
.

**Fig. 1.**
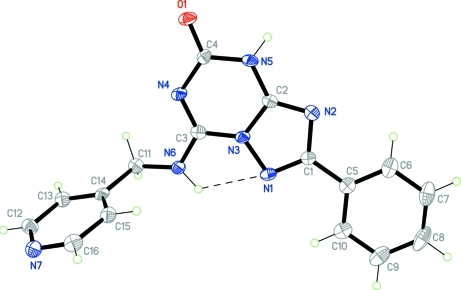
The molecular structure of 2-phenyl-7-(4-pyridylmethylamino)-1,2,4-triazolo[1,5-a][1,3,5]triazin-5-one showing the atomic numbering scheme. Displacement ellipsoids are drawn at the 50% probability level.

**Fig. 2.**
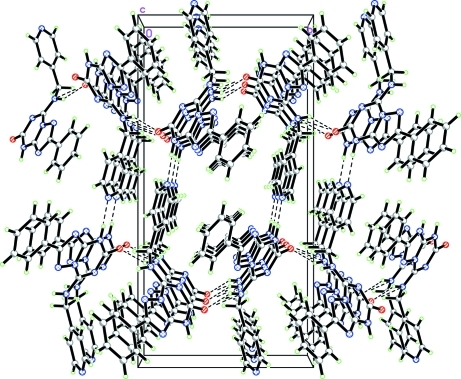
Crystal packing in the cell (view along axis c).

## supplementary crystallographic information

### Comment

The 1,2,4-triazolo[1,5-*a*]triazines are 5-azaisosters of the purine heterocyclic system that carries a bridge nitrogen atom. They have been shown to possess a variety of promising biological effects (Dolzhenko *et al.*, 2006). In continuation of our works on the synthesis and structural investigations of 1,2,4-triazolo[1,5-*a*][1,3,5]triazines (Dolzhenko *et al.*, 2007*a*,*b*; Dolzhenko *et al.*, 2008), we report herein molecular and crystal structure of 2-phenyl-7-(4-pyridylmethylamino)-1,2,4-triazolo[1,5-*a*][1,3,5]triazin-5-one, C_16_H_13_N_7_O. (Fig. 1 & 2).

The 1,2,4-triazolo[1,5-*a*][1,3,5]triazine heterocyclic system is essentially planar with a r.m.s. deviation of 0.0375 Å. The phenyl ring lies practically in the plane of the 1,2,4-triazolo[1,5-*a*][1,3,5]triazine core (with a small dihedral angle of 1.36 (23)° between the mean planes). The amino group nitrogen atom N6 is located 0.2246 (50) Å above the 1,2,4-triazolo[1,5-*a*][1,3,5]triazine mean plane. The pyridine ring is turned out of this plane by twisting of the aminomethyl bridge [C3—N6—C11—C14 torsion angle is 118.50 (40)°]. This results in a dihedral angle of 69.22 (9)° between the 1,2,4-triazolo[1,5-*a*][1,3,5]triazine and pyridine mean planes. The amino group N6H hydrogen atom acts as a hydrogen donor in the NH···N intramolecular hydrogen bonding with the triazole N1 atom.

In the crystal, molecules form a three dimensional network of chains. The *C*(11) chains (Bernstein *et al.*, 1995) parallel to a [100] axis consist of the molecules linked *via* the CONH···N hydrogen bonds between the triazine N6H atom and the N7 atom of pyridine ring. The *C*(6) chains running along [011] and [011] directions are made of the molecules connected *via* the NH···O═C contacts between the N6—H amino group acting as a hydrogen donor and the carbonyl group O1 atom in the role of a hydrogen acceptor.

### Experimental

7-Methylthio-2-phenyl-1,2,4-triazole[1,5-*a*][1,3,5]triazin-5-one (0.52 g, 2 mmol) was added to a solution of 4-pyridylmethylamine (0.32 g, 3 mmol) in DMF (5 ml) and the mixture was heated at 70–80°C with stirring for 6 h. After cooling, ice cold water (40 ml) was added and the product was filtered and recrystalized from methanol. Yield: 0.56 g (87%), m.p. 567 K.

### Refinement

All C-bound H atoms were positioned geometrically and included in the refinement in riding-motion approximation [0.95 Å for aromatic CH and 0.99 Å for methylenic protons; *U*_iso_(H) = 1.2*U*_eq_(C_Ar_) and *U*_iso_(H)= 1.2*U*_eq_(C_methylenic_)] while the N-bound H atoms were located in a difference map and refined freely. 1221 Friedel pairs were merged.

### Crystal data

**Table:**

C_16_H_13_N_7_O	*D*_x_ = 1.451 Mg m^−^^3^
*M**_r_* = 319.33	Melting point: 567 K
Orthorhombic, *P**n**a*2_1_	Mo *K*α radiation, λ = 0.71073 Å
Hall symbol: P 2c -2n	Cell parameters from 569 reflections
*a* = 22.142 (8) Å	θ = 2.6–21.1°
*b* = 11.016 (4) Å	µ = 0.10 mm^−^^1^
*c* = 5.992 (2) Å	*T* = 100 K
*V* = 1461.5 (9) Å^3^	Thin plate, colourless
*Z* = 4	0.58 × 0.36 × 0.04 mm
*F*(000) = 664	

### Data collection

**Table:**

Bruker SMART APEX CCD diffractometer	1830 independent reflections
Radiation source: fine-focus sealed tube	1530 reflections with *I* > 2σ(*I*)
graphite	*R*_int_ = 0.088
φ and ω scans	θ_max_ = 27.5°, θ_min_ = 2.1°
Absorption correction: multi-scan (*SADABS*; Sheldrick, 2001)	*h* = −25→28
*T*_min_ = 0.945, *T*_max_ = 0.996	*k* = −14→14
9607 measured reflections	*l* = −7→7

### Refinement

**Table:**

Refinement on *F*^2^	Primary atom site location: structure-invariant direct methods
Least-squares matrix: full	Secondary atom site location: difference Fourier map
*R*[*F*^2^ > 2σ(*F*^2^)] = 0.067	Hydrogen site location: inferred from neighbouring sites
*wR*(*F*^2^) = 0.145	H atoms treated by a mixture of independent and constrained refinement
*S* = 1.19	*w* = 1/[σ^2^(*F*_o_^2^) + (0.0695*P*)^2^ + 0.135*P*] where *P* = (*F*_o_^2^ + 2*F*_c_^2^)/3
1830 reflections	(Δ/σ)_max_ < 0.001
225 parameters	Δρ_max_ = 0.35 e Å^−^^3^
1 restraint	Δρ_min_ = −0.27 e Å^−^^3^

### Special details

**Table:**

Geometry. All e.s.d.'s (except the e.s.d. in the dihedral angle between two l.s. planes) are estimated using the full covariance matrix. The cell e.s.d.'s are taken into account individually in the estimation of e.s.d.'s in distances, angles and torsion angles; correlations between e.s.d.'s in cell parameters are only used when they are defined by crystal symmetry. An approximate (isotropic) treatment of cell e.s.d.'s is used for estimating e.s.d.'s involving l.s. planes.
Refinement. Refinement of *F*^2^ against ALL reflections. The weighted *R*-factor *wR* and goodness of fit *S* are based on *F*^2^, conventional *R*-factors *R* are based on *F*, with *F* set to zero for negative *F*^2^. The threshold expression of *F*^2^ > σ(*F*^2^) is used only for calculating *R*-factors(gt) *etc*. and is not relevant to the choice of reflections for refinement. *R*-factors based on *F*^2^ are statistically about twice as large as those based on *F*, and *R*- factors based on ALL data will be even larger.

### Fractional atomic coordinates and isotropic or equivalent isotropic displacement parameters (Å^2^)

**Table:**

	*x*	*y*	*z*	*U*_iso_*/*U*_eq_	
O1	0.83275 (13)	0.3791 (3)	0.1382 (6)	0.0251 (8)	
N1	0.78903 (15)	0.0671 (3)	0.7906 (7)	0.0191 (8)	
N2	0.88088 (15)	0.1634 (3)	0.7854 (7)	0.0186 (8)	
N3	0.79295 (15)	0.1475 (3)	0.6125 (6)	0.0174 (8)	
N4	0.76558 (15)	0.2401 (3)	0.2791 (7)	0.0187 (8)	
N5	0.86084 (16)	0.2819 (3)	0.4502 (6)	0.0186 (8)	
H5N	0.900 (2)	0.301 (4)	0.429 (8)	0.018 (12)*	
N6	0.70697 (15)	0.0862 (3)	0.4338 (6)	0.0201 (8)	
H6N	0.712 (2)	0.025 (5)	0.544 (10)	0.039 (15)*	
N7	0.48227 (17)	0.1748 (3)	0.3615 (7)	0.0236 (8)	
C1	0.84184 (19)	0.0814 (4)	0.8851 (8)	0.0177 (9)	
C2	0.84876 (18)	0.2014 (3)	0.6159 (7)	0.0166 (9)	
C3	0.75455 (18)	0.1596 (3)	0.4347 (7)	0.0175 (9)	
C4	0.81929 (18)	0.3037 (4)	0.2826 (8)	0.0183 (9)	
C5	0.85949 (19)	0.0149 (4)	1.0875 (7)	0.0199 (9)	
C6	0.9147 (2)	0.0359 (4)	1.1868 (8)	0.0264 (10)	
H6	0.9422	0.0910	1.1197	0.032*	
C7	0.9305 (2)	−0.0226 (4)	1.3837 (9)	0.0329 (12)	
H7	0.9687	−0.0080	1.4504	0.040*	
C8	0.8899 (3)	−0.1029 (4)	1.4827 (9)	0.0358 (13)	
H8	0.9001	−0.1420	1.6190	0.043*	
C9	0.8347 (2)	−0.1258 (4)	1.3827 (9)	0.0317 (11)	
H9	0.8075	−0.1820	1.4492	0.038*	
C10	0.8187 (2)	−0.0678 (4)	1.1872 (7)	0.0241 (10)	
H10	0.7806	−0.0832	1.1201	0.029*	
C11	0.66773 (18)	0.0743 (4)	0.2433 (7)	0.0206 (10)	
H11A	0.6682	−0.0112	0.1928	0.025*	
H11B	0.6838	0.1248	0.1202	0.025*	
C12	0.49957 (19)	0.1204 (4)	0.1724 (8)	0.0242 (10)	
H12	0.4698	0.1032	0.0627	0.029*	
C13	0.55916 (19)	0.0879 (4)	0.1287 (8)	0.0218 (10)	
H13	0.5698	0.0500	−0.0082	0.026*	
C14	0.60260 (19)	0.1119 (4)	0.2896 (8)	0.0187 (9)	
C15	0.58514 (19)	0.1699 (4)	0.4811 (8)	0.0211 (9)	
H15	0.6141	0.1894	0.5923	0.025*	
C16	0.5247 (2)	0.2002 (4)	0.5120 (8)	0.0245 (10)	
H16	0.5133	0.2406	0.6456	0.029*	

### Atomic displacement parameters (Å^2^)

**Table:**

	*U*^11^	*U*^22^	*U*^33^	*U*^12^	*U*^13^	*U*^23^
O1	0.0245 (16)	0.0180 (14)	0.0328 (19)	0.0027 (12)	0.0069 (15)	0.0101 (15)
N1	0.0230 (18)	0.0146 (17)	0.0196 (17)	−0.0022 (14)	0.0025 (17)	0.0034 (15)
N2	0.0180 (18)	0.0137 (16)	0.0240 (18)	0.0024 (13)	0.0013 (16)	−0.0026 (16)
N3	0.0192 (17)	0.0115 (16)	0.022 (2)	−0.0018 (13)	0.0019 (15)	0.0038 (15)
N4	0.0214 (18)	0.0105 (16)	0.0241 (19)	0.0029 (13)	0.0030 (16)	0.0038 (16)
N5	0.0176 (18)	0.0123 (17)	0.026 (2)	−0.0018 (13)	0.0039 (16)	0.0019 (16)
N6	0.0173 (18)	0.0153 (17)	0.028 (2)	0.0014 (13)	−0.0002 (17)	0.0088 (17)
N7	0.0244 (19)	0.0164 (17)	0.030 (2)	0.0040 (15)	0.0003 (17)	0.0025 (17)
C1	0.020 (2)	0.0154 (19)	0.018 (2)	0.0020 (16)	0.0032 (18)	−0.0025 (18)
C2	0.018 (2)	0.0092 (17)	0.023 (2)	0.0000 (15)	0.0027 (18)	−0.0024 (18)
C3	0.0169 (19)	0.0130 (19)	0.023 (2)	0.0054 (15)	0.0042 (18)	−0.0031 (18)
C4	0.020 (2)	0.0132 (19)	0.021 (2)	0.0056 (15)	0.0000 (19)	0.0011 (19)
C5	0.025 (2)	0.0156 (19)	0.019 (2)	0.0070 (17)	0.0032 (19)	−0.0046 (18)
C6	0.035 (3)	0.022 (2)	0.023 (2)	0.0087 (19)	−0.003 (2)	−0.005 (2)
C7	0.044 (3)	0.024 (2)	0.031 (3)	0.014 (2)	−0.013 (2)	−0.004 (2)
C8	0.062 (4)	0.027 (2)	0.018 (2)	0.022 (2)	−0.006 (2)	−0.002 (2)
C9	0.049 (3)	0.020 (2)	0.026 (2)	0.012 (2)	0.006 (2)	0.004 (2)
C10	0.031 (2)	0.017 (2)	0.024 (2)	0.0049 (17)	0.005 (2)	−0.002 (2)
C11	0.023 (2)	0.0137 (18)	0.025 (3)	0.0005 (17)	0.0012 (19)	−0.0021 (19)
C12	0.022 (2)	0.022 (2)	0.028 (3)	−0.0003 (18)	0.003 (2)	0.000 (2)
C13	0.024 (2)	0.016 (2)	0.025 (2)	−0.0008 (17)	−0.001 (2)	−0.0008 (19)
C14	0.023 (2)	0.0082 (17)	0.025 (2)	0.0013 (15)	0.003 (2)	0.0068 (18)
C15	0.024 (2)	0.015 (2)	0.025 (2)	−0.0018 (17)	−0.004 (2)	−0.0004 (19)
C16	0.034 (3)	0.0121 (19)	0.028 (2)	0.0022 (18)	0.003 (2)	−0.0026 (19)

### Geometric parameters (Å, °)

**Table:**

O1—C4	1.236 (5)	C6—C7	1.389 (7)
N1—C1	1.309 (5)	C6—H6	0.9500
N1—N3	1.389 (5)	C7—C8	1.394 (8)
N2—C2	1.308 (6)	C7—H7	0.9500
N2—C1	1.386 (5)	C8—C9	1.384 (8)
N3—C3	1.370 (5)	C8—H8	0.9500
N3—C2	1.371 (5)	C9—C10	1.381 (7)
N4—C3	1.310 (5)	C9—H9	0.9500
N4—C4	1.380 (5)	C10—H10	0.9500
N5—C2	1.358 (5)	C11—C14	1.526 (6)
N5—C4	1.383 (6)	C11—H11A	0.9900
N5—H5N	0.90 (4)	C11—H11B	0.9900
N6—C3	1.328 (5)	C12—C13	1.392 (6)
N6—C11	1.441 (5)	C12—H12	0.9500
N6—H6N	0.95 (6)	C13—C14	1.387 (6)
N7—C16	1.332 (6)	C13—H13	0.9500
N7—C12	1.338 (6)	C14—C15	1.369 (7)
C1—C5	1.470 (6)	C15—C16	1.392 (6)
C5—C6	1.379 (6)	C15—H15	0.9500
C5—C10	1.415 (6)	C16—H16	0.9500
			
C1—N1—N3	101.5 (3)	C6—C7—H7	120.2
C2—N2—C1	101.8 (3)	C8—C7—H7	120.2
C3—N3—C2	121.9 (4)	C9—C8—C7	120.0 (5)
C3—N3—N1	128.4 (3)	C9—C8—H8	120.0
C2—N3—N1	108.7 (3)	C7—C8—H8	120.0
C3—N4—C4	119.6 (4)	C10—C9—C8	120.6 (5)
C2—N5—C4	120.9 (4)	C10—C9—H9	119.7
C2—N5—H5N	116 (3)	C8—C9—H9	119.7
C4—N5—H5N	120 (3)	C9—C10—C5	119.5 (4)
C3—N6—C11	122.5 (4)	C9—C10—H10	120.2
C3—N6—H6N	110 (3)	C5—C10—H10	120.2
C11—N6—H6N	123 (3)	N6—C11—C14	113.7 (4)
C16—N7—C12	117.7 (4)	N6—C11—H11A	108.8
N1—C1—N2	116.7 (4)	C14—C11—H11A	108.8
N1—C1—C5	122.3 (4)	N6—C11—H11B	108.8
N2—C1—C5	121.0 (4)	C14—C11—H11B	108.8
N2—C2—N5	132.0 (4)	H11A—C11—H11B	107.7
N2—C2—N3	111.3 (4)	N7—C12—C13	123.1 (4)
N5—C2—N3	116.7 (4)	N7—C12—H12	118.5
N4—C3—N6	123.9 (4)	C13—C12—H12	118.5
N4—C3—N3	120.3 (4)	C14—C13—C12	118.5 (4)
N6—C3—N3	115.9 (4)	C14—C13—H13	120.7
O1—C4—N4	122.6 (4)	C12—C13—H13	120.7
O1—C4—N5	117.7 (4)	C15—C14—C13	118.4 (4)
N4—C4—N5	119.7 (4)	C15—C14—C11	123.1 (4)
C6—C5—C10	119.5 (4)	C13—C14—C11	118.5 (4)
C6—C5—C1	120.5 (4)	C14—C15—C16	119.7 (4)
C10—C5—C1	120.0 (4)	C14—C15—H15	120.2
C5—C6—C7	120.8 (5)	C16—C15—H15	120.2
C5—C6—H6	119.6	N7—C16—C15	122.6 (4)
C7—C6—H6	119.6	N7—C16—H16	118.7
C6—C7—C8	119.6 (5)	C15—C16—H16	118.7
			
C1—N1—N3—C3	169.6 (4)	C2—N5—C4—N4	−2.1 (6)
C1—N1—N3—C2	0.9 (4)	N1—C1—C5—C6	−177.5 (4)
N3—N1—C1—N2	−0.4 (5)	N2—C1—C5—C6	2.2 (6)
N3—N1—C1—C5	179.3 (4)	N1—C1—C5—C10	−0.2 (6)
C2—N2—C1—N1	−0.2 (5)	N2—C1—C5—C10	179.5 (4)
C2—N2—C1—C5	−180.0 (4)	C10—C5—C6—C7	−0.3 (6)
C1—N2—C2—N5	−179.3 (4)	C1—C5—C6—C7	177.1 (4)
C1—N2—C2—N3	0.8 (4)	C5—C6—C7—C8	−0.4 (6)
C4—N5—C2—N2	177.7 (4)	C6—C7—C8—C9	1.3 (7)
C4—N5—C2—N3	−2.5 (6)	C7—C8—C9—C10	−1.4 (7)
C3—N3—C2—N2	−170.7 (3)	C8—C9—C10—C5	0.6 (7)
N1—N3—C2—N2	−1.1 (4)	C6—C5—C10—C9	0.2 (6)
C3—N3—C2—N5	9.4 (5)	C1—C5—C10—C9	−177.2 (4)
N1—N3—C2—N5	179.0 (3)	C3—N6—C11—C14	−118.5 (4)
C4—N4—C3—N6	−173.5 (4)	C16—N7—C12—C13	1.1 (6)
C4—N4—C3—N3	6.8 (5)	N7—C12—C13—C14	0.6 (7)
C11—N6—C3—N4	10.2 (6)	C12—C13—C14—C15	−2.0 (6)
C11—N6—C3—N3	−170.1 (3)	C12—C13—C14—C11	179.0 (4)
C2—N3—C3—N4	−11.9 (5)	N6—C11—C14—C15	10.2 (5)
N1—N3—C3—N4	−179.2 (4)	N6—C11—C14—C13	−170.8 (4)
C2—N3—C3—N6	168.4 (4)	C13—C14—C15—C16	1.6 (6)
N1—N3—C3—N6	1.0 (6)	C11—C14—C15—C16	−179.4 (4)
C3—N4—C4—O1	178.6 (4)	C12—N7—C16—C15	−1.5 (6)
C3—N4—C4—N5	0.0 (6)	C14—C15—C16—N7	0.1 (6)
C2—N5—C4—O1	179.1 (4)		

### Hydrogen-bond geometry (Å, °)

**Table:**

*D*—H···*A*	*D*—H	H···*A*	*D*···*A*	*D*—H···*A*
N5—H5N···N7^i^	0.90 (4)	1.89 (4)	2.782 (5)	173 (4)
N6—H6N···O1^ii^	0.95 (6)	1.96 (5)	2.735 (5)	137 (4)
N6—H6N···N1	0.95 (6)	2.31 (5)	2.813 (5)	112 (4)

Symmetry codes: (i) *x*+1/2, −*y*+1/2, *z*; (ii) −*x*+3/2, *y*−1/2, *z*+1/2.
